# Effects of Vagus Nerve Stimulation and Vagotomy on Systemic and Pulmonary Inflammation in a Two-Hit Model in Rats

**DOI:** 10.1371/journal.pone.0034431

**Published:** 2012-04-06

**Authors:** Matthijs Kox, Michiel Vaneker, Johannes G. van der Hoeven, Gert-Jan Scheffer, Cornelia W. Hoedemaekers, Peter Pickkers

**Affiliations:** 1 Department of Intensive Care Medicine, Radboud University Nijmegen Medical Centre, Nijmegen, The Netherlands; 2 Department of Anesthesiology, Radboud University Nijmegen Medical Centre, Nijmegen, The Netherlands; 3 Nijmegen Institute for Infection, Inflammation and Immunity (N4i), Nijmegen, The Netherlands; University of Giessen Lung Center, Germany

## Abstract

Pulmonary inflammation contributes to ventilator-induced lung injury. Sepsis-induced pulmonary inflammation (first hit) may be potentiated by mechanical ventilation (MV, second hit). Electrical stimulation of the vagus nerve has been shown to attenuate inflammation in various animal models through the cholinergic anti-inflammatory pathway. We determined the effects of vagotomy (VGX) and vagus nerve stimulation (VNS) on systemic and pulmonary inflammation in a two-hit model. Male Sprague-Dawley rats were i.v. administered lipopolysaccharide (LPS) and subsequently underwent VGX, VNS or a sham operation. 1 hour following LPS, MV with low (8 mL/kg) or moderate (15 mL/kg) tidal volumes was initiated, or animals were left breathing spontaneously (SP). After 4 hours of MV or SP, rats were sacrificed. Cytokine and blood gas analysis was performed. MV with 15, but not 8 mL/kg, potentiated the LPS-induced pulmonary pro-inflammatory cytokine response (TNF-α, IL-6, KC: p<0.05 compared to LPS-SP), but did not affect systemic inflammation or impair oxygenation. VGX enhanced the LPS-induced pulmonary, but not systemic pro-inflammatory cytokine response in spontaneously breathing, but not in MV animals (TNF-α, IL-6, KC: p<0.05 compared to SHAM), and resulted in decreased pO_2_ (p<0.05 compared to sham-operated animals). VNS did not affect any of the studied parameters in both SP and MV animals. In conclusion, MV with moderate tidal volumes potentiates the pulmonary inflammatory response elicited by systemic LPS administration. No beneficial effects of vagus nerve stimulation performed following LPS administration were found. These results questions the clinical applicability of stimulation of the cholinergic anti-inflammatory pathway in systemically inflamed patients admitted to the ICU where MV is initiated.

## Introduction

Patients with severe sepsis and septic shock often suffer from respiratory failure, placing them in need of mechanical ventilation (MV). While this intervention is lifesaving, MV can lead to, or worsen lung injury; a condition called ventilator-induced lung injury (VILI) [1]. MV-induced cyclic stretch and/or overinflation elicits a pulmonary inflammatory response (biotrauma) characterized by cytokine production, neutrophil recruitment and lung edema, resulting in impaired lung function [2], [3]. Patients with systemic inflammation, such as in sepsis, are at increased risk for developing VILI [4]. It is thought that the additional inflammatory insult, or second ‘hit’ induced by MV, synergizes with the underlying systemic inflammatory process, resulting in detrimental effects on the lungs and other organs (multiple-hit theory) [5], [6]. This cascade of events can ultimately lead to multiple organ dysfunction syndrome (MODS), associated with high mortality [7]. In clinical practice, treatment aimed to limit the initial overwhelming systemic pro-inflammatory state in sepsis has not proved very successful [8]. However, limiting the second inflammatory hit caused by MV may represent a viable therapy to reduce lung injury and subsequent multi-organ failure in systemically inflamed patients.

Recently, it was demonstrated that the vagus nerve can reflexively limit the innate immune response via binding of its neurotransmitter acetylcholine to the α7 nicotinic acetylcholine receptor (α7nAChR) present on macrophages [9], [10]. This mechanism was named ‘the cholinergic anti-inflammatory pathway’. Electrical stimulation of the vagus nerve has shown to attenuate inflammation and improve outcome in several animals models [11]–[13]. However, this pathway has been relatively sparsely studied in VILI. We have recently shown that pre-treatment with the selective α7nAChR agonist GTS-21 ameliorates VILI in mice [14]. In addition, two very recent investigations demonstrated that electrical vagus nerve stimulation attenuates VILI induced by injurious tidal volumes [15], and in a two-hit model (hemorrhagic shock followed by MV) [16], while transection of the vagus nerve (vagotomy) resulted in worse outcome [16]. However, in these studies, stimulation or ablation of the cholinergic anti-inflammatory pathway was performed before the initial insult [14], [15], or twice, before the first and second insult [16], thereby limiting its clinical applicability.

In the present study we investigated the effects of vagus nerve stimulation and vagotomy on systemic and pulmonary inflammation using a two-hit model in rats. We used lipopolysaccharide (LPS) administration as the first and MV using low and moderate tidal volumes as the second hit, to model the sepsis patient that is admitted to the ICU in a hyperinflammatory state where MV is initiated. As mitigating the initial pro-inflammatory hit is often not possible in clinical practice, we applied vagus nerve stimulation or vagotomy after LPS administration and before the start of mechanical ventilation.

## Materials and Methods

### Reagents

Lipopolysaccharide (LPS, derived from *E. coli*, serotype 0111:B4) was obtained from Sigma-Aldrich (St Louis, MO, USA) and was dissolved in 0.9% NaCl. The LPS solution was sonicated for a minimum of 30 minutes prior to use. Alfaxalone was purchased from Vétoquinol (Buckingham, UK).

### Animals

All procedures described were in accordance with the requirements of the Dutch Experiments on Animals Act, the EC Directive 86/609 and approved by the Animal Ethics Committee of the Radboud University Nijmegen Medical Center (permit no. RU-DEC 2010-068). Male Sprague-Dawley rats (Charles River, Sutzfield, Germany) weighing 300–450 gram were used in all experiments. All surgery was performed under alfaxalone anesthesia, and all efforts were made to minimize suffering.

### Experimental protocol

**Figure 1:**
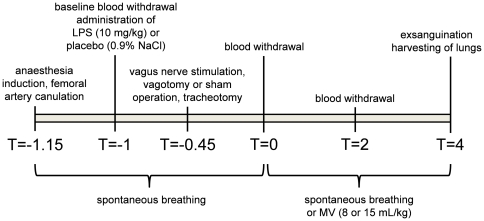
Experimental procedures.

The tail vein was cannulated for anesthesia induction and maintenance. Anesthesia was induced by infusion of alfaxalone (15 mg/kg) and maintained by continuous infusion of 33 mg/kg/hour alfaxalone throughout the experiment. The right femoral artery was cannulated to allow continuous blood pressure monitoring, Ringer's lactate administration (5 mL/kg/hour, containing 4 IE/ml heparin) and blood sampling. LPS (10 mg/kg) or placebo (0.9% NaCl) was administered via the tail vein (time-point T = −1). Next, a cervical midline incision was made and the left vagus nerve was exposed. In sham-animals (SHAM), the wound was covered with moist gauze for 10 minutes. In vagus nerve stimulation animals (VNS), the vagus nerve was prepared free from the carotid artery and, without transecting the nerve, placed on a bipolar platinum electrode (Plastics One, Roanoke, VA, USA) connected to a Grass S11 stimulator with a SIU5 stimulus isolation unit. The nerve was stimulated for 3 minutes at 5 V, 5 Hz, 2 ms [17], [18]. In vagotomy animals (VGX), both the left and right vagus nerves were transected (bilateral vagotomy). Subsequently, rats were tracheotomized with and the tracheal cannula was fixed with ligatures. Animals were left spontaneously breathing using a nose cone supplying air with an FiO_2_ of 40% until 1 hour after LPS/placebo administration when mechanical ventilation (MV) was started (T = 0). Animals were ventilated using a Ugo Basile UB7025 ventilator (Hugo Sachs Elektronik-Harvard Apparatus, March-Hugstetten, Germany). Ventilation parameters: tidal volume 8 or 15 mL/kg, frequency 75 (8 mL/kg) or 40 (15 mL/kg) breaths/min, PEEP 1 cmH_2_O, FiO_2_ 40%. Spontaneously breathing (SP) animals were not connected to the ventilator but continued breathing air with an FiO_2_ of 40% using a nose cone. Rectal temperature was monitored throughout the experiment and was kept between 36.5°C and 37.5°C using a heating pad and blankets. All animals were sacrificed by exsanguination after 4 hours of mechanical ventilation/spontaneous breathing (T = 4). The experimental protocol is illustrated in Figure 1.

### Blood gas and cytokine measurements

Blood gas parameters pH, pO_2_, pCO_2_, BE, HCO_3_ and lactate were determined with an i-STAT Blood Gas Analyzer (Abbot, Hoofddorp, the Netherlands). EDTA anticoagulated blood was centrifuged for 5 minutes at 14000 rpm and plasma was stored at −80°C until analysis by a simultaneous Luminex assay (Milliplex, Billerica, MA, USA) according to the manufacturer's instructions. Immediately after exsanguination, the lungs were carefully removed. The upper part of the left lung was snap-frozen in liquid nitrogen and stored at −80°C until analysis. Lungs were homogenized in T-PER (Thermo Fisher Scientific, Rockford, IL, USA) supplemented with protease inhibitor (complete EDTA-free tablets, Roche, Woerden, The Netherlands) in M-Tubes using a GentleMACS dissociater (protein_01 program, Miltenyi Biotec, Utrecht, The Netherlands). M-tubes with homogenates were centrifuged (4000 g, 5 min, 4°C), after which the supernatant was transferred to an eppendorf tube and centrifuged again (14000 g, 10 min, 4°C). Cytokines in supernatants were measured by ELISA (Duoset, R&D systems, Minneapolis, MN, USA) according to the manufacturer's instructions.

### Statistical Analysis

Data are expressed as mean±SEM. The Grubbs test (extreme studentized deviate method) was performed and significant outliers were excluded from analysis (maximum of one exclusion per dataset). Statistical differences between groups were detected by one-way ANOVA with Bonferroni post-hoc test. A p-value less than 0.05 was considered statistically significant. All tests were performed with Graphpad Prism 5 (Graphpad Software, La Jolla, USA).

## Results

### MV with 8 mL/kg does not elicit an inflammatory response, nor does it amplify the LPS-induced inflammatory response

**Figure 2:**
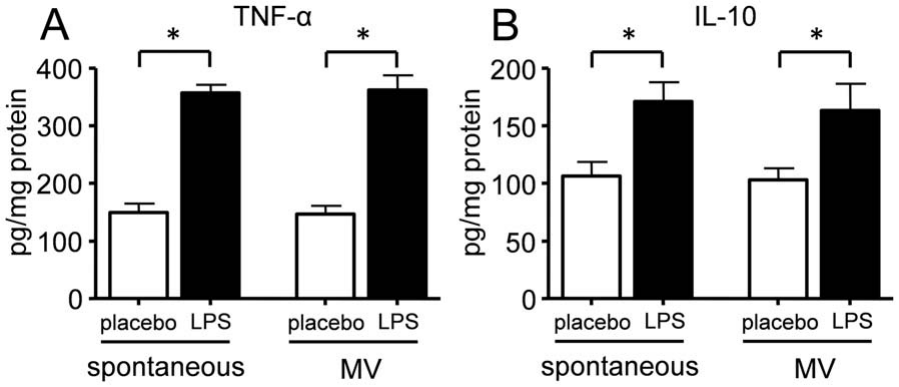
Effects of ventilation with 8 mL/kg in LPS- or placebo-pretreated rats on pulmonary inflammation. Pulmonary concentrations of pro-(A: TNF-α) and anti-inflammatory (B: IL-10) cytokines in placebo- and LPS-treated spontaneously breathing and mechanically ventilated (MV, 8 mL/kg) rats. Pulmonary cytokine levels are normalized to the total amount of protein in each lung homogenate. Data are represented as mean ± SEM of 8–9 animals per group. * indicates p<0.05, one-way ANOVA with Bonferroni post-hoc test.

**Figure 3:**
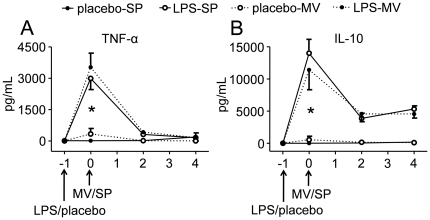
Effects of ventilation with 8 mL/kg in LPS- or placebo-pretreated rats on systemic inflammation. Plasma concentrations of pro-(A: TNF-α) and anti-inflammatory (B: IL-10) cytokines in placebo- and LPS-treated spontaneously breathing (SP) and mechanically ventilated (MV, 8 mL/kg) rats. Data are represented as mean ± SEM of 8–9 animals per group. * indicates p<0.05 between placebo- and LPS-treated groups (one-way ANOVA with Bonferroni post-hoc test). There were no differences between the placebo-SP and placebo-MV or the LPS-SP and LPS-MV groups.

Compared to spontaneously breathing animals, MV with a tidal volume of 8 mL/kg did not result in an increase in pulmonary levels of the pro-inflammatory cytokine TNF-α or the anti-inflammatory cytokine IL-10 (Figure 2). LPS administration in spontaneously breathing animals significantly increased pulmonary cytokine levels. However, MV did not enhance the LPS–induced cytokine response (Figure 2). A similar pattern was observed for other pro-inflammatory cytokines (IL-6 and KC, data not shown). MV did not result in increased levels of plasma cytokines (Figure 3). As expected, LPS administration resulted in high plasma cytokine levels, however, similar to the lung data, MV did not further increase or sustain cytokine levels. Again, this pattern was similar for IL-6 and KC (data not shown).

In placebo-treated animals, MV resulted in increased pH and decreased pCO_2_ compared to spontaneously breathing animals (Table 1). LPS administration resulted in significantly lower arterial blood pressure compared to placebo in both spontaneously breathing and MV animals. Furthermore, the increase in pH observed upon ventilation in placebo-treated animals was not present in LPS-treated animals. In spontaneously breathing rats, LPS administration resulted in lower pCO_2_ levels compared to placebo, probably as a result of inflammation-induced hyperventilation. No differences in oxygenation were observed between any of the groups.

**Table 1:** Cardiorespiratory parameters in placebo and LPS-treated (10 mg/kg) rats ventilated with 8 ml/kg or left breathing spontaneously.

Parameter	group	T = −1	T = 0	T = 2	T = 4
**MAP**	placebo-SP	133±2	98±4^a^	100±8^b^	115±8^d^
	LPS-SP	128±3	71±5^a^	82±4	73±6^d^
	placebo-MV	124±5	97±4	128±7^b,c^	125±9^e^
	LPS-MV	133±3	81±6	91±3^c^	89±6^e^
**pH**	placebo-SP	7.36±0.01	7.31±0.01	7.36±0.02^f^	7.40±0.01^h^
	LPS-SP	7.36±0.01	7.33±0.02	7.35±0.02	7.40±0.02
	placebo-MV	7.34±0.02	7.29±0.01	7.49±0.01^f,g^	7.49±0.02^h,i^
	LPS-MV	7.36±0.01	7.31±0.01	7.40±0.01^g^	7.40±0.02^i^
**pCO_2_**	placebo-SP	59±3	68±3^j^	64±4^l,m^	57±3^n,o^
	LPS-SP	59±5	52±3^j^	51±3^l^	44±4^n^
	placebo-MV	62±3	72±4^k^	38±2^m^	38±1^o^
	LPS-MV	58±1	57±2^k^	43±3	43±4
**pO_2_**	placebo-SP	149±16	122±10	113±10	110±9
	LPS-SP	142±10	126±12	117±10	108±8
	placebo-MV	161±10	134±7	126±7	122±6
	LPS-MV	139±11	141±9	130±9	113±5

LPS/placebo administered at T = −1, mechanical ventilation (MV) started at T = 0. MAP: mean arterial pressure, SP: spontaneously breathing. Data are represented as mean ± SEM of 8–9 animals per group. Superscript character pairs indicate p<0.05, one-way ANOVA with Bonferroni post-hoc test.

### MV with 15 mL/kg does not elicit an inflammatory response, but amplifies the LPS-induced pulmonary inflammatory response

**Figure 4:**
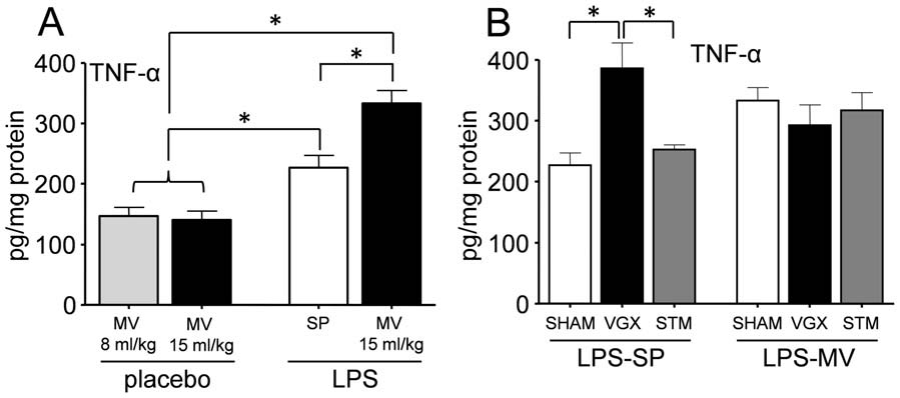
Effects of ventilation with 15 mL/kg in LPS- or placebo-pretreated rats, and vagus nerve stimulation/vagotomy on pulmonary inflammation. A: Pulmonary concentrations of TNF-α in placebo- and LPS-treated spontaneously breathing (SP) and mechanically ventilated (MV, 8 or 15 mL/kg) rats. B: Pulmonary concentrations of TNF-α in LPS-treated spontaneously breathing and mechanically ventilated (15 mL/kg) rats that underwent a sham operation (SHAM), bilateral vagotomy (VGX) or vagus nerve stimulation (STM). Pulmonary cytokine levels are normalized to the total amount of protein in each lung homogenate. Data are represented as mean ± SEM of 6–8 animals per group. * indicates p<0.05, one-way ANOVA with Bonferroni post-hoc test.

Similar to MV with 8 mL/kg, a tidal volume of 15 mL/kg did not result in pulmonary cytokine production (TNF-α shown in Figure 4A). LPS administration in spontaneously breathing animals significantly increased pulmonary cytokine levels, and MV significantly enhanced LPS–induced pro-inflammatory (TNF-α, IL-6 and KC; TNF-α shown in Figure 4A) but not anti-inflammatory (IL-10) pulmonary cytokine levels. MV with 15 mL/kg did not result in increased plasma cytokine levels (data not shown). Similar to the data presented in Figure 3, LPS administration resulted in high plasma cytokine levels, however, MV did not affect plasma cytokine levels (data not shown).

At T = 0 and T = 2 (1 and 3 hours post-administration), both spontaneously breathing and ventilated rats treated with LPS displayed a significantly lower blood pressure compared with placebo-treated ventilated animals (Table 2). At T = 4, blood pressure was restored in LPS-treated spontaneously breathing rats, but still significantly decreased in LPS-MV rats. pH was significantly lower and pCO_2_ significantly higher in spontaneously breathing LPS-treated rats compared with the other groups. Compared with placebo-treated ventilated animals, oxygenation was significantly lower in LPS-treated spontaneously breathing animals. However, no difference in oxygenation between LPS-treated ventilated animals and placebo-treated ventilated animals was found.

**Table 2:** Cardiorespiratory parameters in placebo and LPS-treated (10 mg/kg) sham-operated rats ventilated with 15 ml/kg or left breathing spontaneously.

Parameter	group	T = −1	T = 0	T = 2	T = 4
**MAP**	placebo-MV	96±6	113±5^a,b^	128±6^c,d^	113±5^e^
	LPS-SP	102±0	92±6^a^	102±5^c^	102±5
	LPS-MV	102±2	90±4^b^	92±4^d^	81±6^e^
**pH**	placebo-MV	7.35±0.03	7.32±0.03	7.58±0.01^f^	7.55±0.02^h^
	LPS-SP	7.37±0.01	7.33±0.02	7.34±0.01^f,g^	7.40±0.02^h,i^
	LPS-MV	7.34±0.01	7.32±0.01	7.54±0.03^g^	7.53±0.03^i^
**pCO_2_**	placebo-MV	63±5	68±6	30±1^j^	32±2^l^
	LPS-SP	61±3	60±3	57±3^j,k^	51±3^l,m^
	LPS-MV	65±3	58±3	30±8^k^	30±2^m^
**pO_2_**	placebo-MV	144±22	139±15	198±3^n^	199±5^o^
	LPS-SP	133±17	164±3	143±4^n^	135±3^o^
	LPS-MV	182±20	162±13	172±21	166±22

LPS/placebo administered at T = −1, mechanical ventilation (MV) started at T = 0. MAP: mean arterial pressure, SP: spontaneously breathing. Data are represented as mean ± SEM of 6 animals per group. Superscript character pairs indicate p<0.05, one-way ANOVA with Bonferroni post-hoc test.

### Vagotomy enhances pulmonary inflammation and impairs oxygenation in spontaneously breathing, but not in ventilated (15 mL/kg) rats

Vagotomy resulted in significantly increased pulmonary levels of all pro-inflammatory cytokines in LPS-treated spontaneously breathing animals compared with sham-operated or vagus nerve-stimulated rats (TNF-α depicted in Figure 4B). IL-10 was not affected by vagotomy (data not shown). Furthermore, vagotomy exerted no effects in ventilated rats (Figure 4B). Plasma cytokine levels were not affected by vagotomy in spontaneously breathing or ventilated LPS-treated animals (data not shown). In ventilated animals, no effects of vagotomy on blood pressure or blood gas parameters were found (data not shown). However, in spontaneously breathing rats, vagotomy resulted in significantly lower blood pressure at T = 0 and lower pO_2_ levels at T = 0 and T = 2 compared to sham-operated animals (Table 3). Vagus nerve stimulation affected neither pulmonary (TNF-α depicted in Figure 4B) or plasma cytokine levels (data not shown), nor blood gas parameters in both spontaneously breathing (Table 3) and ventilated LPS-treated animals (data not shown).

**Table 3:** Cardiorespiratory parameters in LPS-treated (10 mg/kg) spontaneously breathing rats that underwent a sham operation, vagotomy, or vagus nerve stimulation.

Parameter	group	T = −1	T = 0	T = 2	T = 4
**MAP**	SHAM	102±0	92±6^a^	102±5	102±5
	VGX	101±6	74±2^a^	103±5	99±8
	STM	96±4	87±5	99±7	91±9
**pH**	SHAM	7.37±0.01	7.33±0.02	7.34±0.01	7.39±0.02
	VGX	7.38±0.03	7.33±0.02	7.36±0.02	7.38±0.01
	STM	7.37±0.02	7.32±0.02	7.33±0.03	7.40±0.02
**pCO_2_**	SHAM	61±3	60±3	57±3	51±3
	VGX	57±6	54±3	48±1	48±2
	STM	59±4	59±3	58±4	49±4
**pO_2_**	SHAM	133±17	164±3^b^	143±4^c^	135±3
	VGX	124±20	116±13^b^	93±11^c^	105±4
	STM	124±22	147±14	128±11	118±13

LPS administered at T = −1. MAP: mean arterial pressure, SHAM: sham operation, VGX: vagotomy, STM: vagus nerve stimulation. Data are represented as mean ± SEM of 6 animals per group. Superscript character pairs indicate p<0.05, one-way ANOVA with Bonferroni post-hoc test.

## Discussion

In this study, we show that mechanical ventilation with moderate, but not with low tidal volumes potentiates the pulmonary inflammatory response elicited by systemic LPS administration. Vagotomy applied after LPS administration resulted in amplification of the LPS-induced pulmonary inflammatory response and reduced oxygenation in spontaneously breathing animals. However it did not affect inflammatory or respiratory parameters in ventilated rats. Vagus nerve stimulation performed after LPS administration had no effects in either ventilated or spontaneously breathing rats.

In the absence of LPS, MV with low (8 mL/kg) or moderate (15 mL/kg) tidal volumes did not result in a pulmonary or systemic inflammatory response. In contrast, we [14], [19] and others [20] have recently shown that 4–5 hours of MV with 8 mL/kg in mice results in increased levels of inflammatory cytokines in both lungs and plasma. It appears that rats are less susceptible to MV-induced pulmonary inflammation. For instance, 4-hour ventilation with 8 mL/kg did not result in increased levels of pulmonary cytokines or neutrophils [6]. Even MV with 24 mL/kg did not lead to increased wet/dry ratios or impaired oxygenation, while only moderate increases in pulmonary inflammation were found [6]. This probably accounts for the common use of very high tidal volumes (>30 cmH_2_O) in rat VILI models that hinder interpretation with regard to the human situation [21]–[23]. These findings signify that there are large interspecies differences with regard to the inflammatory response to MV. With respect to humans, a recent study in ARDS patients demonstrated that lowering of tidal volumes from 6.3 to 4.2 mL/kg accompanied by extracorporeal carbon dioxide removal was associated with a significant decrease in inflammatory mediators in bronchoalveolar lavage fluid [24], suggesting that there is no clear cutoff value for safe tidal volumes in humans.

The second hit, MV, potentiated the first hit induced by systemic LPS administration with regard to pulmonary inflammation, but only when a moderately high tidal volume of 15 mL/kg was used. This tidal volume might still be clinically relevant in light of the partially collapsed or fluid-filled ‘baby lungs’ [25] observed in septic patients with lung injury of which only a portion is ventilated [16]. We chose to start MV one hour after LPS administration. At this time-point, high plasma concentrations of TNF-α, an important cytokine in the pathophysiology of VILI [26], are found. Using this approach, we attempted to simulate a patient with systemic hyperinflammation admitted to the ICU for initiation of mechanical ventilation. While we did find increased levels of all pro-inflammatory cytokines in lungs of LPS-treated ventilated animals compared with LPS-treated spontaneously breathing rats, we could not demonstrate a statistically significant impaired gas exchange. In this respect, our two-hit model does not represent a true lung injury model. The direct interaction of systemic LPS administration and MV has been scarcely studied. The most commonly used two-hit models employ intratracheal LPS administration or hemorrhagic shock followed by MV [16], [27]–[29]. Both of these treatments are expected to have a more severe effect on lung function compared with systemic LPS administration which might explain the lack of an effect on gas exchange in our study. In a rat study where LPS was also i.v. administered one hour before the start of 4-hour MV, increased lung inflammation and injury was found in LPS-treated ventilated rats compared with non-ventilated non-LPS-treated rats [30]. Unfortunately, no spontaneously breathing LPS-treated or placebo-treated MV groups were studied for comparison [30]. Two similar studies in rabbits have investigated direct interactions between systemic LPS and MV using moderate tidal volumes (10–15 mL/kg) [31], [32]. However, in these studies, LPS was administered 2 hours after the start of a 6–8 hour MV period. Nevertheless, the authors did find impaired oxygenation, altered histopathological lung alterations and reduced compliance in LPS-treated MV animals compared with a spontaneously breathing LPS-treated group or placebo-treated MV group. In line with our results, these studies also demonstrated that MV potentiates the pulmonary, but not systemic inflammatory response to i.v. LPS administration [31], [32].

Our result partly corroborate previously described findings of increased pulmonary inflammation after vagotomy [16]. In spontaneously breathing rats, vagotomy enhanced LPS-induced pulmonary inflammatory cytokine levels and was associated with impaired oxygenation, suggestive of a direct inhibitory effect of the vagus nerve on pulmonary cytokine production. However, no effects were observed in LPS-treated MV rats. These seemingly contradictory results have led us to formulate an alternative hypothesis. Pulmonary stretch receptors play an important role in mediating information on lung mechanics via the afferent vagus nerve, and it was recently shown that inhibition of afferent vagus nerve signaling by cooling resulted in increased tidal volumes [33]. Therefore, it could be speculated that, in our experiments, vagotomy abrogated the pulmonary stretch receptor-feedback mechanism, leading to increased, injurious tidal volumes and subsequent elevated pulmonary cytokine levels in spontaneously breathing animals. In this regard, it is of note that in our experiments, a clearly noticeable change in breathing pattern was observed after bilateral vagotomy. This would also explain why no effects of vagotomy on pulmonary cytokine production were observed in mechanically ventilated rats, where breathing pattern and tidal volume were controlled by the ventilator.

It was recently demonstrated that vagus nerve stimulation attenuates the pulmonary inflammatory response and improves oxygenation in rats subjected to hemorrhagic shock and subsequent ventilation with 12 mL/kg for 4 hours [16]. In contrast, we found no effects of vagus nerve stimulation on any of the studied parameters. One might argue that we have stimulated the nerve for a too short period (3 minutes). This appears not to be a plausible explanation however, as in mice, the anti-inflammatory effects of vagus nerve stimulation were equal whether it was performed for 0.5, 2 or 20 minutes [18]. Therefore, the lack of an effect is likely due to the timing of the intervention. In the aforementioned study, vagus nerve stimulation was performed prior to induction of hemorrhagic shock, hence before the ‘first hit’ [16]. We stimulated the vagus nerve following the first hit but before initiation of MV; in our view a more clinically relevant time-point. The inflammatory cascade induced by LPS administration might have been too far advanced for vagus nerve stimulation to limit it. However, this does not explain why stimulation did not attenuate the MV-induced increase in pulmonary inflammation elicited by LPS. Nevertheless, studies that reported beneficial results of vagus nerve stimulation on inflammation have performed stimulation before or simultaneous to the inflammatory stimulus/insult [10]–[13], [15]–[18], [34]–[36]. Interestingly, a very recent investigation describes the effects of vagus nerve stimulation performed 4 hours after induction of polymicrobial sepsis by cecal ligation and puncture (CLP) and found no reduction of lung injury or pulmonary and systemic inflammatory markers [37]. In agreement, the acetylcholinesterase inhibitor physostigmine, which reinforces the cholinergic anti-inflammatory pathway by increasing ACh availability, attenuated inflammation and septic shock when administered before CLP induction, but not when administered 6 hours afterwards [38], indicating that the timing of stimulation of the cholinergic anti-inflammatory pathway is critical.

In conclusion, this study demonstrates no beneficial effects of vagus nerve stimulation on systemic or pulmonary inflammation in a two-hit model in rats where stimulation was performed at a clinically relevant time point: following the initial hit but before initiation of MV. This questions the clinical applicability of stimulation of the cholinergic anti-inflammatory pathway in systemically inflamed patients admitted to the ICU where MV is initiated.
